# Viral Hepatitis E and Chronicity: A Growing Public Health Concern

**DOI:** 10.3389/fmicb.2020.577339

**Published:** 2020-09-29

**Authors:** Vikram Thakur, Radha Kanta Ratho, Swatantra Kumar, Shailendra K. Saxena, Ishani Bora, Pryanka Thakur

**Affiliations:** ^1^Department of Virology, Post Graduate Institute of Medical Education and Research, Chandigarh, India; ^2^Centre for Advanced Research, Faculty of Medicine, King George’s Medical University, Lucknow, India

**Keywords:** hepatitis E virus, chronicity, genotypes, immunocompromised, organ transplants, ribavirin

## Abstract

Hepatitis E viral infection recently emerges as a global health concern. Over the last decade, the understanding of hepatitis E virus (HEV) had changed with the discovery of new genotypes like genotype-7 and genotype-8 with associated host and mode of infection. Diversification in the mode of hepatitis E infection transmission through blood transfusion, and organ transplants in contrast to classical feco-oral and zoonotic mode is the recent medical concern. The wide spectrum of infection ranging from self-limiting to acute liver failure is now overpowered by HEV genotype-specific chronic infection especially in transplant patients. This concern is further escalated by the extra-hepatic manifestations of HEV targeting the central nervous system (CNS), kidney, heart, and pancreas. However, with the development of advanced efficient cell culture systems and animal models simulating the infection, much clarity toward understanding the pathogenetic mechanism of HEV has been developed. Also this facilitates the development of vaccines research or therapeutics. In this review, we highlight all the novel findings in every aspect of HEV with special emphasis on recently emerging chronic mode of infection with specific diagnosis and treatment regime with an optimistic hope to help virologists and/or liver specialists working in the field of viral hepatitis.

## Introduction

Hepatitis E virus (HEV) is one of the leading causes of acute viral hepatitis (AVH) with worldwide distribution. According to the World Health Organization (WHO) factsheet (2015), there have been 20 million HEV infections annually causing 3.3 million symptomatic cases with ∼60,000 deaths due to viral hepatitis (Hakim et al., 2017; Nimgaonkar et al., 2018). Over the last decade, the clinical and basic research on HEV pathogenesis has gone to such an extent that the European Association for the Study of the Liver (EASL) has recently released the clinical practice guidelines on viral hepatitis E in 2018 (Dalton et al., 2018). Presently in developing countries, approximately 30% of the populations are being infected with HEV with mortality ranging from 0.2 to 1% in the general population. Around 1–4% of AVH in the general population and 30% of acute liver failure (ALF) in pregnant women are attributed to viral hepatitis E (Purcell and Emerson, 2008; Khuroo, 2011). About 50% of acute hepatitis, 30–45% of ALF and 30–70% sporadic hepatitis cases in the Indian subcontinent are being attributed to infection (Kumar Acharya et al., 2007; Aggarwal and Jameel, 2011) whereas 27–80% of HEV seroprevalence has been observed in the general population in SE-Asian countries (Abe et al., 2006).

Traditionally HEV infection considered as acute, self-limited, and endemic to developing countries including the Indian subcontinent, however, progression to chronicity, resulting in liver damage and cirrhosis, has been reported in immunocompromised patients with solid organ transplant (SOT), human immunodeficiency virus (HIV) and hematological malignancies (Kamar et al., 2013a; Murali et al., 2015). This review comprehensively describes the least understood extra-hepatic manifestation of HEV infection, i.e., chronic hepatitis E in-relation to susceptibility in different genotypes, clinical profiling of patients with special emphasis on the treatment regimen in SOT recipients with chronic HEV infection.

## Emerging HEV Genotypes and Predilection Toward Chronicity

**FIGURE 1:**
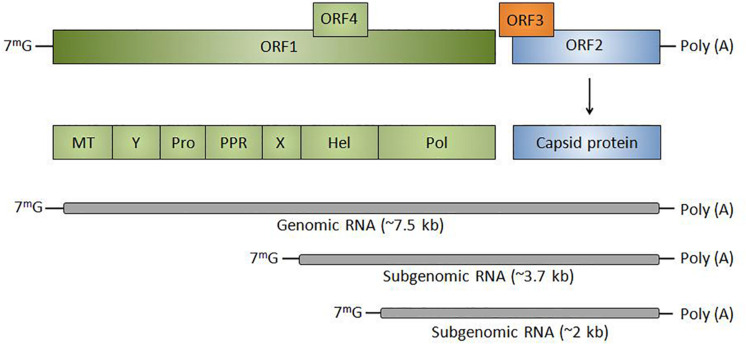
Genomic organization of HEV: Classically divided into three ORFs (ORF1, ORF2, and ORF3) except genotype-1 having an additional ORF4. HEV RNA is transcribed into subgenomic RNA of ∼3.7 and ∼2 kb. The 5′ non-coding region is capped with 7-methylguanosine (7mG) and 3′ is polyadenylated [poly(A)]. ORF1 encodes for non-structural proteins including MT, regions of unknown function (Y, PPR, and X), Pro, Hel, and Pol or RdRp. ORF2 codes for capsid protein and ORF3 is a multifunctional protein that helps in the release of virions from the infected cell.

Hepatitis E virus is a positive-sense, single-stranded RNA virus with a 7.2 kb genome, divided into three open reading frames (ORFs) except genotype-1 having an additional ORF4 (Figure 1; Balayan et al., 1983). The 5′ non-coding region is capped with 7-methylguanosine (7mG) and 3′ is polyadenylated [poly(A)]. Open reading frame 1 (ORF1) encodes for a polyprotein of ∼190 KDa comprising of non-structural proteins including regions of unknown function [Y, proline-rich region (PRR), and X] and methyltransferase (MT), cysteine protease (Pro), helicase (Hel) and RNA polymerase (Pol). ORF2 and ORF3 are translated from a subgenomic RNA of 2.2 Kb where ORF2 encodes for capsid protein which becomes N-glycosylated at three sites. The unique ORF4 encodes a protein that stimulates viral RNA-dependent RNA-polymerase (RdRp) and promotes viral replication. The life cycle of HEV includes various crucial steps that initiate from the attachment of HEV to the heparin sulfate proteoglycans followed by clathrin-mediated endocytosis and release of viral RNA into the cytoplasm. The viral RNA encodes for ORF1 protein followed by replication via negative-strand RNA intermediated, synthesis of full-length and subgenomic RNAs. Subgenomic RNAs undergo translation that yields ORF2 and ORF3 proteins followed by packaging, assembly, and release of the newly generated virus. Pieces of evidence are suggesting that ORF3 is associated with the release of HEV into the bloodstream (Debing et al., 2016a).

**FIGURE 2:**
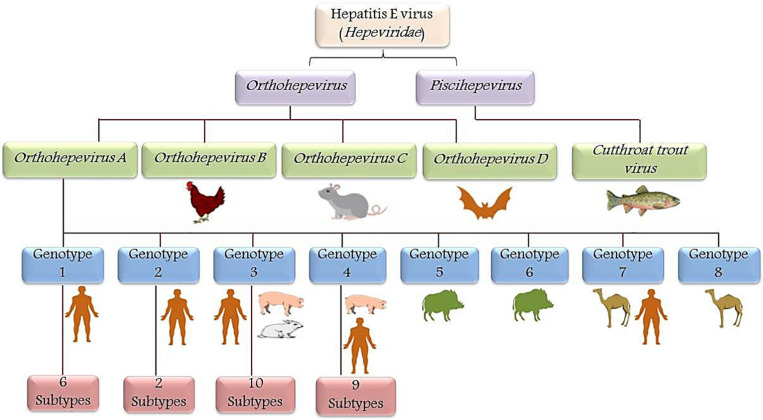
Schematic representation of HEV taxonomy: Hepeviridae family is classified into genera *Orthohepevirus* and *Piscihepevirus*. *Orthohepevirus* is comprised of four species viz. *Orthohepevirus-A*, *Orthohepevirus-B*, *Orthohepevirus-C*, and *Orthohepevirus-D* based on the host range and sequence identities, whereas *Piscihepevirus* genus includes only the *Cutthroat trout virus* (CTV). In *Orthohepevirus-A* species, eight genotypes were identified and designated as HEV genotype (HEV-1 to HEV-8). Genotype-1 (six subtypes) and genotype-2 (two subtypes) are mainly confined to humans. However, genotype-3 (10 subtypes) and genotype-4 (9 subtypes) infects swine but are also known to be transmitted to humans by the consumption of meat (zoonotic). Genotype-5 and genotype-6 are identified in wild boars. Recently genotype-7 and genotype-8 were identified in the dromedary and Bactrian camel, respectively.

Recently eight phylogenetically distinct genotypes (genotypes 1–8) within a single HEV serotype have been identified with four genotypes (genotype 1–4) known to cause human infection (Figure 2). All the isolated HEV strains from the Indian subcontinent showed 82% sequence similarity whereas; the Mexican strain showed 77% sequence similarity across the entire genome, thereby suggested the possibility of two different genotypes, i.e., HEV genotype-1 and genotype-2 (Smith et al., 2016). Later, a novel HEV genotype-3 stain with 79–80% sequence similarity was isolated from domestic swine (Meng et al., 1997). Whereas, HEV genotype-4 has been discovered in the sera of a Chinese patient with acute hepatitis (Wang et al., 1999). Predominantly, HEV transmission is by the fecal-oral route in endemic areas (genotype-1 and genotype-2), whereas food-borne transmission is common in non-endemic developed countries (genotype-3 and genotype-4) (Zhou et al., 2004). HEV-5 and HEV-6 have only been identified in wild boars (Tei et al., 2003). A recent discovery of camelid HEV in dromedary camels from Dubai in 2014 and Bactrian camels from Xinjiang in 2016 with more than 20% nucleotide difference proposed HEV genotypes-7 and genotype-8, respectively (Hoofnagle et al., 2012). Based on the analysis of nucleotide p-distances, HEV-1 was identified to have six subtypes, whereas HEV-2, 3, and 4 have 2, 10, and 9 subtypes, respectively (Smith et al., 2016).

Among the existing eight genotypes, genotype-1 is more virulent and responsible for high mortality in pregnant women and genotype-3 has been exclusively reported in chronic hepatitis E. However a case of persistent HEV infection with genotype-4 was also reported in a child (Geng et al., 2014; Kamar et al., 2012b). Similarly, HEV RNA and antigen was also detected in a woman with nephritic syndrome infected with chronic HEV genotype-4 (Geng et al., 2016). A very few case reports have also been reported where transplant recipient patients were infected with HEV genotype-1. So far, there is no chronic case reported with HEV genotype-2, 5, 6, and 8.

Key factors which might be the player in the causation of chronic infection are as follows:

1. HEV chronic cases are mostly reported with genotype-3 and genotype-4 and one case with genotype-7, as these cases are mostly reported from the developed countries with an increase in meat consumption pointing toward the zoonotic mode of transmission of HEV chronicity.
2. Immunocompromised patients with an impaired state of immunity have been identified as the high-risk individuals developing chronic HEV infection.
3. High virulence in addition to the endemic nature of HEV genotype-1 strain might further increase the chances of chronic cases.

Following is a brief review of the HEV chronic cases reported in the organ transplant recipient immunocompromised patients.

## Chronicity in Immunocompromised Organ Transplant Recipients

**FIGURE 3:**
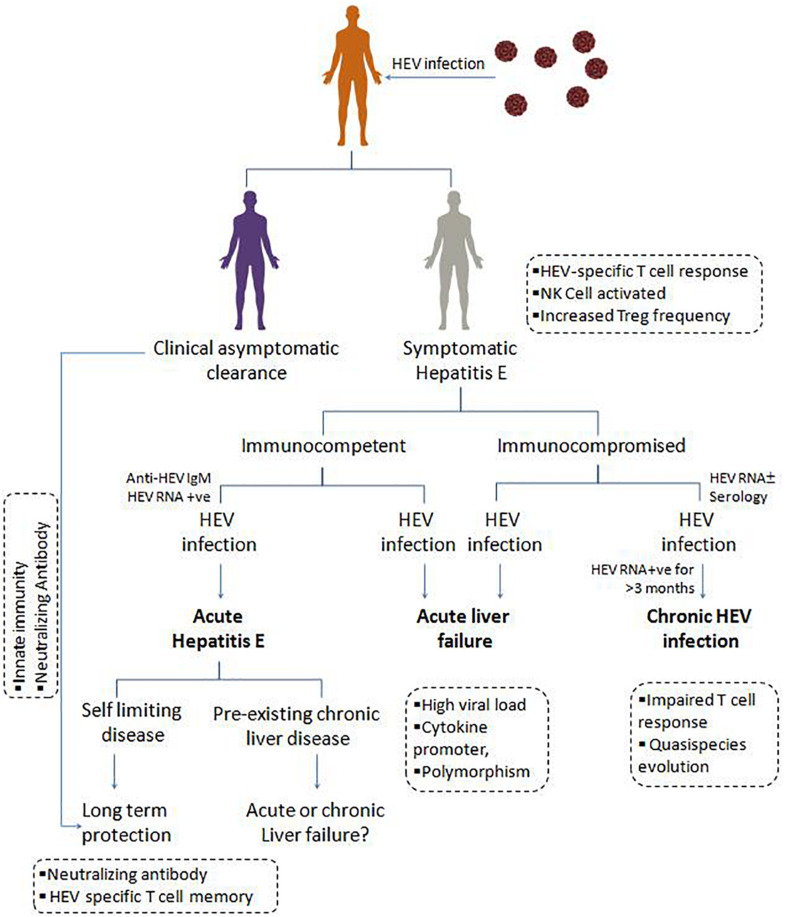
Clinical manifestations, and immune response in HEV infection: On contact with the hepatitis E virus, individuals may undergo asymptomatic clearance owing to innate immunity and neutralizing antibody. Symptomatic immunocompetent and immunocompromised individuals with hepatitis E viral infection present as acute hepatitis E, which is usually a self-limiting form of disease providing long term protection due to HEV specific T cell memory and neutralizing antibody. Acute liver failure due to high HEV load and associated inflammatory cytokines. In immunocompromised patients, the presence of HEV RNA for more than 3 months, results in chronic HEV infection, possibly attributed by the quasi-species evolution in HEV and impaired T cell response.

Generally, immunocompetent individuals develop a self-limiting acute HEV infection which eventually subsides (Figure 3). However in immunocompromised SOT patients (Kamar et al., 2014a), over 60% of AVH infection progress to chronicity, and 10% under-goes cirrhosis within 2 years (Haagsma et al., 2009; Kamar et al., 2011a; Lhomme et al., 2012). Generally, chronic hepatitis E infection is asymptomatic and causes liver damage when it progresses to cirrhosis, and eventually, patients succumb to death. Such patients are potent sources for HEV transmission due to prolonged viremia and viral shedding in feces, i.e., for more than 3 months. Since the first case of chronic HEV infection reported in 2008 in liver transplant patients (Unzueta and Rakela, 2014), to date, many studies reported the immunopathology, clinical implication, and treatment regimen for these critically ill patients. Also, kidney, heart, and pancreas transplant recipients with lymphoma and leukemia are more likely to progress to chronicity (Kamar et al., 2008; Europe PMC, 2020). So, in such immunocompromised individuals, chronic hepatitis E has now emerged as an important clinical problem that needs to be addressed.

So far, the majority of the chronic HEV cases have been seen with genotype-3 except for few reports of persistent infections observed with genotype-1 and 4 (Geng et al., 2014). The basic human pathogenetic mechanisms behind chronicity in immunocompromised state and SOTs are not well understood, however, the immuno-suppressants might be playing some role.

In a remarkable study, the progression of 8 of the 14 HEV infected SOT recipients have been reported to chronicity which was inclusive of all three liver transplant recipients (Kamar et al., 2008). This suggests that HEV chronicity is most common among liver transplant recipients and is observed in low frequency across the globe (Kamar et al., 2011a; Unzueta and Rakela, 2014). Similarly, a case of HEV chronic hepatitis leading to progressive cirrhosis and death in a liver transplant recipient has been reported to receive a liver from an occult HEV infected donor (Schlosser et al., 2012). Similarly in a prospective study on 287 orthotopic liver transplant recipients (OLT), four chronic HEV infected patients with enlarged densely infiltrated portal tracts with interface hepatitis were identified (Galante et al., 2015). A 3-month short regimen with ribavirin (400–800 mg/day) is effective in these patients. Agarwala et al. (2018) reported the absence of HEV viremia in a cohort of 30 liver transplant recipients till the 6 months post-transplant follow-up, despite 20% seropositivity. Similarly, a cohort of studies by Naik et al. (2013) and Munjal et al. (2014) demonstrated no viremia in a cohort of 205 renal transplant recipients since transplantation and no detectable HEV RNA in 49 renal transplant recipients, respectively. These observations suggest to us the limited potential of the circulating HEV genotype-1 to cause chronic infections in the Indian population.

Interestingly a unique case of chronic HEV infection by genotype-7 was reported by Lee et al. (2016) in a liver transplant recipient which probably due to the consumption of camel meat and milk. In 2020 Japanese nationwide study of HEV infection on 99 heart and 2526 kidney transplant recipients revealed HEV RNA positivity in 1 heart (1.01%) and 11 (0.44%) kidney transplant recipients with the dominance of genotype-3, wherein four patients developed chronic hepatitis E post-transplantation (Owada et al., 2020). Chronic HEV might explain many cases of otherwise unexplained post-transplant hepatitis or cryptogenic cirrhosis. A case report by Kurihara et al. (2016) described a 41-year-old patient who had cirrhosis of the liver due to non-alcoholic steatohepatitis and hepatocellular carcinoma before liver transplant. However post-liver transplant, the recipient was infected with HEV through blood transfusion later progressed to chronicity and was also treated with ribavirin therapy (800 mg/day for 20 weeks). Halac et al. (2012a) studied a cohort of 14 children who had undergone OLT and found that 86% of them had anti-HEV IgG post-OLT. However, one case presented with anti-HEV IgG, IgM, and HEV RNA in different samples collected during and after 8 years of OLT. The most unusual finding revealed was the presence of two different phylogenetically established HEV strains (genotype-3) causing re-infection leading to chronic hepatitis which eventually progresses to fibrosis and cirrhosis.

Waldenström et al. (2015) reported chronic HEV infection by genotype-3 in a 63-year-old Swedish heart transplant recipient who received blood from the 17 donors. A long-term follows up of HEV infection in 446 renal transplant recipients were studied by Sridhar et al. (2018), where four patients (0.9%) developed anti-HEV IgM, of them three progressed to chronic hepatitis E with HEV RNA positivity which was attributed to consumption of undercooked pig products and genotype-4 was confirmed by phylogenetic analysis. They were on prednisolone and everolimus as immunosuppressive prophylaxis, however, later treated with ribavirin (400 mg twice daily). A novel HEV-3 strain (SW/16-0282) showing 87.8% homology with HEV genotype-3h TR19 strain was also reported from kidney transplant recipient with chronic hepatitis E from Switzerland (Wang et al., 2017). In six heart transplant recipients, on immunosuppression with tacrolimus and prednisolone combination, elevated liver enzymes with RNA positivity by HEV genotype-3 were reported (Koning et al., 2013). The first case of chronic HEV genotype-4, in the renal transplant recipient, was reported from China (Wang et al., 2018). A unique case of a 33-year-old man with ileocolonic Crohn’s disease, acquired chronic HEV due to genotype-1, following a trip to India. This rare case was treated by reducing the doses of prednisolone, followed by a 24-week course of ribavirin (600 mg twice daily) which lowered the HEV viral load. This case has important implications for travelers who are immunocompromised and are traveling to HEV endemic areas of the world (Robins et al., 2018). Recently, a case of chronic hepatitis E with genotype-1 was reported in a post liver transplant patient with previous compensated cryptogenic cirrhosis (Rathi et al., 2020), necessarily warranting to focus on chronic hepatitis E of different genotypes in different countries.

## HEV Chronicity and Extrahepatic Manifestations

**FIGURE 4:**
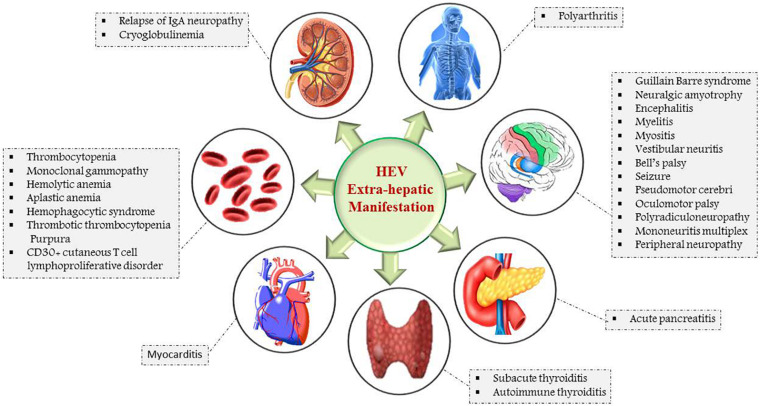
Schematic representation of extra-hepatic manifestations of HEV infection: Myocarditis and acute pancreatitis are the HEV associated manifestations of heart and pancreas. The major neurological and hematological manifestations are Guillain Barre syndrome, Bell’s palsy, neuralgic amyotrophy, thrombocytopenia, hemolytic, and aplastic anemia. Cryoglobulinemia is attributed by renal, whereas polyarthritis is associated with skeletal-related HEV manifestations.

Hepatitis E infection has been associated with a wide spectrum of extra-hepatic, mainly neurological, renal, cardiac, and hematological manifestations (Fousekis et al., 2020; Figure 4). HEV also has been detected in the human placenta, breast milk, and urine (Pischke et al., 2017; Rivero-Juarez et al., 2019).

Neurological: HEV is associated with neuronal damage in the form of Guillain-Barre syndrome (GBS), neuralgic amyotrophy (NA), encephalitis, myositis, and Bell’s palsy (Dalton et al., 2016). More than 150 cases with HEV genotype-3 have been reported from Europe. However, the majority (∼90%) of the documented cases are immuno-competent which is different from immunocompromised organ transplant recipients. Prevalence of acute hepatitis E in patients with GBS was found to be in the range of 4–8% in various studies, i.e., Netherlands (5%), Japan (4.8%), and Belgium (8%) (Stevens et al., 2017). From a European multicentric study involving 118 NA patients, an unusual clinical presentation like bilateral involvement and damage to brachial plexus and phrenic nerve in patients with HEV associated NA was observed by Van Eijk et al. (2017). Interestingly, HEV RNA could also be detected in cerebrospinal fluid (CSF) of them indicating the central nervous system (CNS) involvement. A case of HEV infected male with high liver function tests (LFTs) and a triad of bilateral shoulder pain has also been reported in single centric research (Dalton and Seghatchian, 2016). Besides, 5.5% (7/126) of the patients with acute and chronic HEV infection had developed neurologic manifestations in a study by Kamar et al. (2011b). Moreover, four immunocompromised patients had chronic hepatitis E infection with genotype-3, of which three were SOT recipients, while one had HEV-HIV co-infection. All the infected patients had neuronal damage such as encephalitis, peripheral demyelinating polyneuropathy, and associated cognitive dysfunction.

Renal manifestations like membranoproliferative glomerulonephritis and cryoglobulinemia are common with HEV infected patients. Membranoproliferative glomerulonephritis and IgA nephropathy relapses were noted in SOT recipients with acute and chronic HEV infection (Kamar et al., 2012a). Similarly, Ali et al. (2001) documented membranoproliferative glomerulonephritis and membranous glomerulonephritis in both immunocompetent and immunosuppressed patients, infected with HEV genotype-1 and genotype-3, respectively. HEV RNA has been also detected in cryoprecipitate obtained from HEV infected cryoglobulinaemic glomerulonephritis patients (Guinault et al., 2016). A case of nephrotic syndrome associated with chronic HEV infection was reported in a kidney transplant recipient, which was completely resolved after the introduction of ribavirin therapy (Taton et al., 2013).

Hematological manifestations like different patterns of anemia (hemolytic, autoimmune hemolytic, and aplastic) (Leaf et al., 2017) have been reported during HEV infection. Severe thrombocytopenia has been described in patients with acute HEV genotype-1 and genotype-3 infections. In 25% of the patients infected with HEV genotype-3, monoclonal paraprotein has been documented (Woolson et al., 2014).

Rheumatologic manifestations such as arthralgia, myalgia, skin rashes, and cryoglobulinemia have been reported in chronic HEV infected liver transplant recipients (Pischke et al., 2014). In a study with solid organ recipient suffered from HEV, 52.9% prevalence of cryoglobulinemia was reported (Marion et al., 2019).

## HEV Chronicity in Co-Infections and Hematological Malignancies

Hepatitis E virus chronic infection has also been reported in patients who were already affected by hepatitis B virus (HBV), hepatitis C virus (HCV), HIV, and chronic liver diseases. Studies have shown that HEV super-infection in patients with chronic liver disease (CLD) leads to liver decompensation.

The first case of dual chronic HEV-HIV infection was reported by Dalton et al. (2009) in a 48 years old male patient having low (<200) CD4 count and abnormal transaminases with pieces of evidence of cirrhosis and inflammation on liver biopsy. The development of cirrhosis was found to be the main concern in HIV/HEV co-infection. Despite anti-retroviral treatment, HEV RNA in serum persisted for 18 months, possibly due to severe immunosuppression and low CD4 count. The prevalence of chronic HEV-HIV dual infection is <0.5% from Europe and Australia, where the cases had consistently low CD count of <200 (Colson et al., 2011; Kaba et al., 2011; Jagjit Singh et al., 2013; Yong et al., 2014). Among the 3000 HIV positive patients, one had chronic HEV infection reported from the United States (Kuniholm et al., 2016). Further studies are hence needed to address whether infection or exposure to HEV plays a role in the development of liver fibrosis in HIV-infected individuals. Co-infections with hepatotropic viruses including HCV and HBV have been described to mediate flares of liver disease causing more damage (Sagnelli et al., 2014). HEV super-infection in 10 CLD patients has also been identified by Monga et al. (2004), of which five were alcoholics, two were cryptogenic, and HBV positive whereas one was positive for HCV. Such varied co-infections might suppress immunity leading to chronic HEV.

Cancer patients especially, hematological are on heavy immunosuppression, therefore frequently prone to other infections. HEV genotype-3 chronicity in four French chronic lymphocytic leukemia (CLL) patients who were earlier exposed to ibrutinib and rituximab have been reported (Open Forum Infectious Diseases Oxford Academic, 2020). This hematological complication was treated successfully with the administration of ibrutinib and ribavirin (median 600 mg ribavirin daily, 8 mg/kg) combination, considering the anemic status. Despite on ribavirin therapy for 3–9 months, three patients were succumbed to death due to underlying disease, while one continued on ibrutinib. Oilier et al. (2009) reported a case of chronic hepatitis E in a 77-year-old male with non-Hodgkin’s lymphoma who was on rituximab. Usually, a 3-month course of ribavirin induces sustained HEV reduction in patients with hematological malignancies (Tavitian et al., 2015). In a Philadelphia chromosome-positive acute lymphoblastic leukemia (ALL) patient, HEV reactivation and viremia till fourteen weeks after allogeneic stem cell transplant (SCT) has been reported (Le Coutre et al., 2009). This reactivation may be attributed to immunosuppression or ALL relapse, however, these consequences are common in patients receiving stem cell transplantation and chemotherapy (Tavitian et al., 2010). In a retrospective study on 328 allogeneic hematopoietic SCT recipients, 8 (2.4%) HEV RNA positive and five chronic hepatitis cases with four deaths have been reported (Versluis et al., 2013). In a child recovered from acute leukemia, the progression of chronic HEV genotype-1 infection toward CLD has been found (NCBI, 2020). In hematological malignancies, the development of cirrhosis has been described in pediatrics marrow transplant patients within 2 years of infection (Halac et al., 2012b).

## Chronicity in Pregnant Women

In pregnant women, the rate of mortality is around 25%, primarily due to fulminant hepatic failure. High perinatal mortality is associated with obstetric complications like preeclampsia and hemorrhage (Kamar et al., 2012b). In pregnancy, chronic hepatitis is stated as epiphenomenal due to temporary perturbation of immunity. In developing countries, high mortality in pregnant women was attributed to virulent HEV genotype-1 strain. However, only a few cases were reported in developed countries due to acute HEV genotype-3 (Mallet et al., 2013; Tabatabai et al., 2014). A case of chronic autochthonous HEV genotype-3c infected pregnant women, who were on immunosuppressive agents for ulcerative colitis (infliximab and azathioprine) was reported (Charre et al., 2018). Despite high HEV load (6.9 log10 copies/ml), the vertical transmission was not reported in the baby, indicating that chronic HEV-3 infection might resolve after pregnancy due to reduced T-cell response and innate immunity. From the study of chronic HEV in a pig model (Cao et al., 2017) Th1/Th2 imbalance has been proposed as the crucial host factor mediating progression toward HEV chronicity during the immunocompromised state.

## HEV Infection and Chronicity in Pediatric and Young Population

Hepatitis E virus infection in children and the pediatric population is usually asymptomatic; however, the symptoms might mimic with viral hepatitis A which is supposed to be common in children and younger age groups. Symptomatic HEV infection is uncommon in children even during large HEV epidemics. Since it is uncommon in the pediatric age group, so also the ALF which reflects the paucity of data on chronic HEV in Childhood (Fischler et al., 2016).

The literature showed the positive relationship of HEV seropositivity with advancing age (Meng et al., 2015). Few studies available indicate low HEV seropositivity in the pediatric age groups (5%) under the 10 years (Bayhan et al., 2016) in Turkey and Morocco whereas 4.2% positivity was observed in children between 2 and 18 years in the province of Van, Turkey (Atabek et al., 2004). Even in a large worldwide survey in early childhood revealed HEV seroprevalence in less than 10% in children up to 10 years of age and less than 5% in European children (Verghese and Robinson, 2014). The Dutch pediatric study found a seroprevalence of 3.2% in liver/kidney transplant patients in contrast to immunocompetent patients with 7.4% positivity (Hoerning et al., 2012). In a retrospective study from France on 96 children who had undergone liver transplants, eight patients (8.3%) were HEV seropositive with one reported case of chronic cytolysis.

Mexican study on 99 pediatric patients, revealed the presence of HEV genotype-3 with 3 and 6%, HEV IgG, and HEV IgM seropositivity, respectively (López-Santaella et al., 2020). In Japan, 3.3% of children with acute HEV hepatitis and 2.6% of the general population are HEV IgG positive (Okano et al., 2020). Conditions like Thalassemia facilitate HEV related chronicity, thereby increased the risk of morbidity as evidenced by Abdelmawla et al. (2019), where anti-HEV IgG and HEV IgM positivity was 24.29 and 2.86%, respectively, among thalassemic children. Similarly, studies from Scandinavia (Psichogiou et al., 1996) and Saudi Arabia (Al-Fawaz et al., 1996) showed 2.4 and 10.7% HEV antibody seroprevalence in thalassemic patients respectively. In Germany, 4.6% of anti-HEV IgG prevalence is reported in the pediatric population (Buti et al., 2008), whereas liver transplant children had 3.2–15% seroprevalence (Halac et al., 2012a). In general chronic HEV is seen with less than 2% cases of transplants. One pediatric patient developed HEV-induced liver cirrhosis after hematological stem cell transplantation (Halac et al., 2012a). In an isolated cohort study of 90 pediatric renal allograft recipients had 13.3%, (12/90) HEV IgG positivity with 4.4% (4/90) recipients had HEV genotype-3 active replication and further leading to chronicity (Cordts et al., 2018). Thus from the available world literature, it is evident that children less frequently get affected with HEV and seldom develop chronicity.

## Hepatitis E as a Cause of Acute-On-Chronic Liver Failure

Acute-on-chronic liver failure (ACLF) is an acute event associated with 50–90% mortality in general and 70% in HEV-ACLF cases. HEV acting as a potential trigger of ACLF is a matter of great concern with relevant clinical implications. The severity of liver damage may be associated with the infecting genotype. HEV genotype-3 has been reported to induce ACLF in elderly men or patients with underlying CLD (Péron et al., 2007; Kumar and Saraswat, 2013). Studies from China and the Indian subcontinent (Oilier et al., 2009), reveal a far worse outcome with HEV genotype-1 or 2, however, composite mortality was reported as up to 67% with a median of 34% (Kumar and Saraswat, 2013). ACLF manifestations range from acute deterioration of liver functions to ascites, hepatic encephalopathy, and/or hepatic coagulopathy. Blasco-Perrin et al. (2015) identified 3% (*n* = 11/343) patients with decompensated liver disease associated with acute HEV infection and three deaths in the British/French population. From an Indian study on 368 cases of ACLF, a 12% incidence rate for HEV associated infections was reported but mortality in such cases was lower in comparison to other ACLF etiologies (Shalimar Kedia et al., 2017a, b). In West African countries, ACLF is not common due to acute HEV infection (Shimakawa et al., 2016). Jagadisan et al. (2012) studied a subset of 36 children with CLD superimposed with an acute hepatic insult due to HEV in 27 (75%) children and observed that 17 of them are under ACLF category. High seroprevalence of anti-HEV antibody in a case-control study involving 109 patients with CLD indicating association between HEV infection and advanced stage of CLD has been identified (Kondili et al., 2006). HEV is considered as one of the common causes of ACLF in patients with chronic HBV infection in HEV endemic areas. Zhang et al. (2017) observed 60% HEV infected patients with underlying liver diseases specifically with chronic HBV infection (40%), lead to more severity. In a kidney transplant recipient from France, 47% of chronification has been reported with overt hepatitis E infection (Lee et al., 2016). Generally, 20–50% of transplant recipients following HEV infection develop chronic infection. According to the study by Gupta and Lama (2017), acute HEV infection accounts for over 20% of ACLF cases in Asian countries.

As per “*Asia Pacific Association for Study of Liver*” (APASL), ACLF is defined as “acute hepatic insult manifesting as jaundice (bilirubin >5 mg/dl) and coagulopathy (International normalized ratio, INR >1.5), complicated within 4 weeks by ascites and/or hepatic encephalopathy in a patient with previously diagnosed or undiagnosed chronic liver disease” (Sarin et al., 2019). Superadded HEV infection is a common cause of ACLF in India (Ramachandran et al., 2004; Kumar et al., 2008). A study by Gawande et al. (2019), on 208 patients with ACLF showed a 13.94% contribution by viral hepatitis of which 7.2% exclusively with HEV which acts as precipitating factor for ACLF.

In chronic HBV infected patients, HEV super-infection has an enormous contribution toward ACLF progression (El Sayed Zaki and Othman, 2011). Li et al. (2020) observed that 34.7% (67/193) chronic HBV infected patients had developed ACLF following acute HEV infection and 44.8% (30/67) of them had a poor prognosis. This suggests that HEV super-infection with genotype-3 upon HBV or underlying diseases is a precipitating event to initiate ACLF in patients. Other studies from around the world describing the impact of HEV infection as CLD or/and ACLF manifestations were described in Table 1.

**TABLE 1:** Presenting the cases/studies around the world, i.e., India, China, Nepal, Pakistan, United States, France, and Egypt, etc., where HEV manifests as an acute-on-chronic liver failure (ACLF) and/or chronic liver disease (CLD).

Country/city	Population	Cases of decompensating due to HEV infection (%)
India, New Delhi (Kumar Acharya et al., 2007; Kumar et al., 2009; Garg et al., 2012)	1. Cirrhotic patients with liver decompensation, CHD = 31, ACLF = 42	6 (19.3%) 21 (50%)
	2. ACLF = 91	14 (15.3%)
	3. ACLF = 48	7 (14.5%)
India, Chandigarh (Duseja et al., 2010; Lal et al., 2011; Duseja et al., 2013)	1. ACLF = 100 2. ACLF = 102 3. ACLF = 31	8 (8%) 4 (3.9%) 3 (9.6%)
India, Lucknow (Radha Krishna et al., 2009; Jagadisan et al., 2012)	1. ACLF = 121 2. ACLF (pediatrics) = 36	80 (66.1%) 23 (63.8%)
India, Vellore (Ramachandran et al., 2004)	1. ACLF patients = 9	9 (100%)
China, Guangzhou (Ke et al., 2006; Zhang et al., 2010)	1. ACLF = 107 2. CHB/HEV = 136	80 (74.7%) 54 (39.7%)
China, Shanghai (Zhang et al., 2017)	1. ACLF = 301	34 (11.3%)
Nepal, Kathmandu (Kc et al., 2006, 2009)	1. Cirrhosis with hepatic decompensation = 12	12 (100%)
	2. ACLF = 7	7 (100%)
Bangladesh, Dhaka (Mahtab et al., 2006, 2009)	1. Cirrhosis with hepatic decompensation = 32	14 (43.75%)
	2. ACLF = 69	15 (21.7%)
Pakistan, Karachi (Hamid et al., 2002)	1. ACLF = 4	4 (100%)
France (Toulouse) (Péron et al., 2007) (Villejuif) (Haim-Boukobza et al., 2015)	1. Fulminant hepatitis failure = 7 2. Acute alcoholic hepatitis = 84	7 (100%) 3 (3.5%)
United States (Houston) (Kyvernitakis et al., 2015) (Bethesda) (Fontana et al., 2016)	1. HCV cirrhotic with cancer = 47 2. ACLF = 681	None 3 (0.4%)
Egypt, Mansoura (El Sayed Zaki and Othman, 2011)	1. ACLF = 100	13 (13%)

## Animal Models for Chronic Hepatitis E

To elucidate the pathogenetic mechanism, the reason behind high severity in pregnant patients associated with genotype-1, chronicity in transplant recipients and to develop an effective vaccine, permissive, and highly efficient cell culture system and/or suitable animal model is the utmost requirement (Table 2).

**TABLE 2:** Animal and cell culture models used for the propagation of different genotypes of Hepatitis E virus.

Animal model	Infected with HEV strain	Outcome	References
**Rabbit**			
Rabbit	Swine genotype-4 (intraperitoneally)	Viral shedding in stool, blood, spleen	Wu et al., 2017
Rabbit	Wild-boar derived genotype-3	Viral RNA in liver and gall bladder	Schlosser et al., 2019
Rabbit (pregnant)	KOR-Rb-1	Elevated TNF-α, IFN-γ, histopathology shows necrosis	Ahn et al., 2017
Rabbit	Homologous CHN-BJ-rb14	Prolonged viremia and fecal shedding. Histopathology shows portal fibrosis	Han et al., 2014
Rabbit	Rabbit HEV	RNA from brain, heart, lungs, placenta, kidney	Wang et al., 2017
Rabbit (HEV vaccine evaluation)	HEV 239 vaccine	Induce good immune response	Zhang et al., 2015
Rabbit	Recombinant rat HEV capsid	Protection from HEV genotype-3	Schlosser et al., 2019
Rabbit	P179 vaccine candidate Genotype 4 (strain H4-NJ703)	Protection from HEV	Cheng et al., 2012
Rabbit	Human genotype-4	Viral shedding in stool, elevated LFTs	Ma et al., 2010
**Rat**			
Athymic nude rat (intravenous)	Rat HEV strain LA-B350	Viral RNA in stool, serum 4 × 10^6^ copies/ml	Debing et al., 2016b
**Mouse**			
Balb/c mice	Rabbit strain of HEV genotype-3	Viremia and shedding in feces	Sun et al., 2018
Balb/c nude mice	Swine feces-derived HEV genotype-4	HEV antigen and RNA in kidney, spleen, liver	Huang et al., 2009
Balb/c mice	Full-length swine HEV cDNA clone of genotype-4	HEV RNA in serum, colon, liver	Yu et al., 2018
Humanized FRG mice (intrasplenic inoculation)	HEV genotype-3 stool suspension	Viral RNA in feces	Sayed et al., 2017
Humanized FRG mice	Genotype-1 strain (Sar-55)	RNA in blood 10^4^ IU/ml	Sayed et al., 2017
uPA-SCID mice	HEV genotype-3 Kernow C1-P6	Viral RNA in stool, 6.2 × 10^4^ IU/ml	Sayed et al., 2016
uPA-SCID mice (intrasplenic inoculation)	Genotype-1 strain (Sar-55)	Viremia and HEV RNA in stool	Sayed et al., 2016
uPA^+/+^Nod-SCID-IL2Ry^–/–^	Fecal derived HEV genotype-3	Viremia	van de Garde et al., 2016
**Chicken**			
Chicken	HEV genotype-1	Infectious virus in egg white	Guo et al., 2007
**PIG**			
Pig	Rabbit HEV	Viremia and fecal virus shedding	Cossaboom et al., 2012
Pig	Capsid antigens from rat and chicken HEV strains	Strong anti-HEV IgG response in pigs, protection against HEV genotype-3	Sanford et al., 2012
**Non-human primates**			
Cynomolgus macaques	Chinese rabbit HEV isolate	Elevated LFT’s and shedding in feces	Liu et al., 2013
Cynomolgus macaques	HEV genotype-8 strain from Bactrian camels	Fecal shedding and viremia	Wang et al., 2019
Cynomolgus macaques	HEV genotype-3 strain	Mild elevation of liver enzymes, viremia, and fecal shedding	Gardinali et al., 2017

**HEV CELL CULTURE MODELS**			

**Hepatoma cell lines**			

HepG2/C3A cells	Semi-purified HEV GT3 Kernow-C strain	Permissive cell line with 7.5-fold higher foci count	Shukla et al., 2011
PLC/PRF/5 cell	HEV genotype-1	Efficient cell culture system supporting HEV replication	Takahashi et al., 2010
PLC/PRF/5 cell and A549	Genotype-4 HE-JF5/15F strain	Efficient cell culture system supporting HEV replication Final 3.9 × 10^8^ copies/ml	Tanaka et al., 2009
**Non-hepatoma cell lines**			
2BS cells	87A GT1 HEV strain	Cytopathic effects with viral RNA	Huang et al., 1995
Oligodendrocytic cell line M03.13	Genotype-3 replicon based on Kernow-C1/p6 strain	Support replication of the modified HEV GT1 strain Sar55 pSK-E2/S17	Drave et al., 2016
JEG-3	HEV genotype-1 and 3	Support production of infectious particles	Knegendorf et al., 2018
**Primary cells**			
Primary human hepatocytes (PHHs)	Swine derived HEV genotype-3 and 4	High number of cells in foci	Oshiro et al., 2014

So far, human lung carcinoma (A549) and human Hepatoma (PLC/PRF/5) cell lines have shown better permissibility for HEV, replicative efficiency, and viral load till seven passages (Emerson et al., 2004). Suitable animal models are imperative to understand the immunopathogenesis of Chronic HEV.

In the Western countries, HEV genotype-3 infections are predominant in immunocompromised patients due to zoonotic mode of transmission. To simulate the chronic course of the disease, a rabbit model (Japanese white rabbits) has been developed by inoculating HEV-3 (CH-BJ-rb14 strain) intravenously and detected HEV RNA (positive and negative strands) in kidney along with HEV ORF3 protein by immunohistochemistry, thereby confirmed the replication inside the renal tissues (Wang et al., 2017). Hematoxylin and Eosin (H&E) staining of the liver portal area and renal interstitial sections showed infiltration of inflammatory cell, fibrosis, and infiltration of lymphocytes and plasma cells, respectively, and indicated the scope of simulating chronic hepatitis infection in the animal model. Another *in vivo* chimeric mouse model (urokinase-type plasminogen activator; uPA) mimicking chronic HEV genotype-3 was developed by van de Garde et al. (2016), This small but novel model was prepared by using plasma/fecal samples from eight HEV genotype-3 infected immunocompromised patients to examine the HEV genotype-3 infectivity in samples of different clinical origins and also to develop 100% chronic HEV infection. Similar humanized liver mouse models for chronic HEV-1 and HEV-3 were also developed (Sayed et al., 2016). Small mouse models were easy to handle and care, but are not a natural host of HEV. However, the large mammal-like Cynomologus monkey represents the close ancestor relationship with humans. HEV infection has been successfully induced in Cynomolgus monkeys using HEV RNA-positive urine samples and unravels the potential relationship between HEV infection and kidney disease (Geng et al., 2016). However, due to large size and other ethical issues, relatively small and easy to handle animals have been tried for developing chronic HEV infection models. In this line, using swine HEV-4 strain, HEV susceptible rabbit model has been developed for chronic HEV-4 infection (Ma et al., 2010).

The most interesting fact is that none of the available models, i.e., mouse, rabbit, or monkey are neither the natural host nor they induce chronic infection with any of the known HEV genotypes (Meng, 2010). Swine/pig is recognized as the major reservoir and important zoonotic mode of HEV-3 and HEV-4 transmission to humans. This correlation indicates that pig could be the best model for studying cross-species infection and mimicking chronic hepatitis E infection in immunocompromised patients. So, an immunosuppression state in a pig model has been successfully developed by infecting with HEV genotype-3 strain and pre-post treatment with cyclosporine, azathioprine, and prednisolone and observed active suppression of HEV specific cell-mediated immune (CMI) responses (Cao et al., 2017). This observation might be the reason facilitating the establishment of chronic HEV infection in the immunosuppressed transplant recipients.

These unique animal models for chronic HEV infection might greatly improve our ability to study HEV infectivity by understanding transmission dynamics and delineate underlying pathogenic mechanisms causing chronicity which might lead to the development of specific and effective antiviral drugs and therapeutics against chronic hepatitis E.

## Determinants/Predictors of Chronicity in HEV Infection

Hepatitis E virus infection is diagnosed as chronic hepatitis when replicative HEV RNA persists in serum/stool until 3–6 months after the original infection (Kamar et al., 2011a). Narayanan et al. (2019) discussed the viral and host factors as determinants of chronicity in HEV infection.

### Viral Factors

The most important factors are viral genotype, zoonotic potential, specificity, and adaptability for a host. HEV genotype-3 is the predominant strain for chronicity with variability showing more clinically apparent disease with genotype 3f then genotype 3c (Lhomme et al., 2012). Few cases of chronic infection with HEV-1 and 4 are reported, but none form HEV-2. Also, the zoonotic potential of HEV-3 is high due to the animal reservoir and zoonotic mode of transmission. Quasi-nature of HEV may be associated with the development of chronicity, due to diversification in the ORF2 region. Re-infections either by two different strains of the same genotype (3c and 3e) or two different genotypes (genotype-3 and 4) have been documented in organ transplant recipients by zoonotic transmission (Moal et al., 2012). Tropism for extra-hepatic organs like CNS, kidney, placenta, and replicative fitness for developing chronic infection may be attributed to short human-sequence insertion in HEV RNA (Shukla et al., 2011). HEV tries to evade innate and adaptive immunity by ORF3 mediated down-regulation of ISG expression and by glycosylation of surface proteins, respectively. The very unusual property of having envelope while circulating in bloodstream as free virions help the virus in efficient replication and also aid in non-specific uptake by different cell types (Feng and Lemon, 2014). These viral factors by different mechanisms increase the propensity for chronic infection in HEV infected patients.

### Host Factors

Host immunity in the form of innate, cell-mediated, or humoral has been playing a crucial role in clearing the infection and protecting the host. Low-level expression of tumor necrosis factor-α (TNF-α) and interleukin-1 receptor agonist (IL-Ra) were reported in organ transplant recipients developing chronic HEV infection. Reduction in natural killer (NK) cell counts explains the possible susceptibility to chronic infection. Significant low count of CD2, CD3, CD4 T cells, and increased serum concentration of chemokines such as regulated upon activation, normal T cell expressed and presumably secreted (RANTES), macrophage inflammatory protein (MIP-1), CXCLB, and monocyte chemoattractant protein-1 (MCP-1) (leukocyte recruiters) were described as predictors of HEV chronicity in organ transplant recipients (Kamar et al., 2008, 2011a). Further in such patients, reduction in the levels of soluble interleukin-2 receptor (IL-2R) reflected the impaired T-cell response. Among the T-helper (Th) arm of the immune response, Th2 response mediates HEV infection toward chronicity by increased secretion of IL-10 (Moal et al., 2012; Suneetha et al., 2012).

The immune-state and nutritional health of the patients also decide the course of HEV infection. Immunocompromised and suppressed state of pregnancy, especially when infected with virulent HEV-1 strain, contributed to severe outcomes possibly by Th2 bias. Serum cytokine levels and micronutrient status also plays a role in the susceptibility (Hoofnagle et al., 2012). Even the type of immunosuppressive regimen is used to influence the likelihood of chronicity (Kamar et al., 2008). Patients on immunosuppression like tacrolimus, everolimus [mammalian target of rapamycin, (mTOR inhibitor)], rituximab (B cell modulator), and methotrexate (TNF-α modulator) are more prone to chronic HEV infection. Other crucial predictors/factors are iatrogenic transmissions especially by infected blood transfusion in an immunosuppressed patient. Therefore testing of solvent treated plasma for the presence of HEV by nucleic acid amplification test (NAT) is of prime significance to avoid chronic HEV infection in the blood recipients. Conclusively, greater HEV quasi-species diversity and impaired immune response collectively seem to play a crucial role in the development of chronic HEV infection.

## Diagnostic and Treatment Regimen for Chronic Hepatitis E

Early diagnosis in any infectious disease is the prime factor to limit the progression, thereby increasing the chances of survival by implementing early treatment interventions. In chronic HEV infection, detection of HEV RNA either in serum and/or in stool samples by reverse transcriptase-polymerase chain reaction (RT-PCR) for a minimum 3–6 month time duration is the reference test for the confirmatory diagnosis (Kamar et al., 2013b). Targeting conserved ORF2 region and using NAT-based HEV RNA assay enhanced the analytical sensitivity and specificity of HEV diagnosis (Baylis et al., 2013). An OD450/630 of >15 in antigen assay is the indicator of chronic hepatitis E and can differentiate with acute infection, (PubMed, 2020) but this does not necessarily represent the presence of infectious virions. Detection of IgG and IgM antibodies against HEV in serum is futile as they are undetectable due to immunosuppression (Pischke et al., 2010). Elevated transaminases usually reflect damage in the liver, however, patients who progressed to chronicity have been found relatively lower transaminases alanine aminotransferase, (ALT) ∼300 vs 1000 IU/L) in comparison to acute infection (Murali et al., 2015). Persistence of HEV RNA between 3 and 6 months in serum/stool samples of HEV acquired transplant recipients has been reported (Kamar et al., 2008). This suggested that the RNA persistence in chronic HEV infections beyond 3 months so, in chronic cases, HEV viral load needs to be detected henceforth to evaluate antiviral and/or immunosuppressive drug response in patients for planning an effective treatment regimen.

Currently, there is no established HEV-specific therapeutic protocol; however antiviral such as ribavirin and interferon like pegylated IFN have been used for the treatment of chronic hepatitis E with some success (Lee et al., 2015; Ding et al., 2017). In 2018, the EASL published guidelines, recommended the use of ribavirin as a standard antiviral for HEV treatment however, the optimal ribavirin treatment regimen is not known (Dalton et al., 2018). It does not increase the risk of rejection, thereby can be used in the patients where immunosuppressive agents cannot be decreased. Ribavirin 5′-monophosphate (active ribavirin) inhibits HEV RNA replication indirectly by inhibiting inosine monophosphate dehydrogenase (IMPDH) and subsequently depleting guanosine-5′-triphosphate (GTP) pool (Paeshuyse et al., 2011). Mallet et al. (2010) studied the use of ribavirin (12 mg/kg/day) in one with idiopathic CD4 T lymphocytopenia and other with dual organ transplant (kidney and pancreas) patient for 12 weeks and observed the clearance of HEV RNA from serum/stool by 4th week of treatment which remained undetectable till 12 weeks follow-up. A CLL patient with chronic hepatitis E infection was treated with the highest recommended dose of ribavirin (1000 mg) and showed sustained virological response (SVR) for 24 weeks (Giordani et al., 2013), however in another report; an SCT patient infected with chronic hepatitis E viral infection was unable to survive, even with high dose (800 mg) of ribavirin (Bettinger et al., 2015). Even from two different studies, different SVR responses were observed with ribavirin in transplant recipient patients (Kamar et al., 2010a, 2014b). In one study, with 600–800 mg ribavirin dose for 3 months protect four recipients with SVR of 6 months, whereas in other studies on 59 transplant patients, 600 mg ribavirin for 3 months provides 78% (SVR 6 months) response. Another study showed the 9/11 SVR in 11 transplant patients who were on ribavirin (600–100 mg) for 5 months (Pischke et al., 2013). In addition to ribavirin therapy, reduction in the dose of immunosuppressive drugs targeting T cells should be the first intervention to help in virus clearance in chronic HEV infected immunocompromised and transplant recipient patients. Pegylated IFN-γ alone and/or in combination with ribavirin has also succeeded in the treatment of chronic HEV-HIV dual infection (Dalton et al., 2011; Jagjit Singh et al., 2013). In a study by Kamar et al. (2010b) administration of 135 mcg/week dose of pegylated IFN-alpha 2a to three liver transplant patients with HEV chronicity for 12 weeks, resulting in clearance of HEV viral RNA in serum and stool. A combined and cumulative effect of reduction in immunosuppressive therapy, interferon therapy, and ribavirin monotherapy for 8 weeks, resulted in the absence of HEV RNA and IgM antibodies in a 55-year-old man infected with chronic HEV genotype-3f post 26 months of OLT (Klein et al., 2015). In two different studies involving SOT and SCT recipients treated with ribavirin, high lymphocyte count is an independent predictor and 66% (9/12) achieved SVR (Kamar et al., 2014b; Tavitian et al., 2015), respectively. Other known antiviral, i.e., sofosbuvir was also reported to show a reduction in HEV viral load but failed to achieve SVR in seven chronic HEV infected patients (Cornberg et al., 2019). Personalized therapy could be the priority of further research. Predictive formula based on host clinical features like underlying disease, type and quantity of immunosuppressive agents, viral load in blood/stool, and liver fibrosis could be considered for patient management.

### Hepatitis E Vaccines

With the concern of graft rejection, immunosuppressant therapy is effective only in 30% HEV patients. In addition to antiviral, the HEV vaccine could be the possible option for treating severe and chronic HEV infection. Hecolin is the first and the only vaccine produced by China, however, it is not approved yet for the commercialization, even though the three doses of the vaccine showed 100% efficacy in phase III clinical trial. With the efficacy of 86.8% up to 4.5 years follow-up, no vaccination associated serious adverse effect during the 12 months follow-up post-vaccine administration was reported (Zhu et al., 2010; Murali et al., 2015). Despite promising results, the safety and efficacy profile in chronic and other vulnerable populations is not clear and needs further studies before recommendations for its worldwide production and use.

### Management of Chronic Hepatitis

**FIGURE 5:**
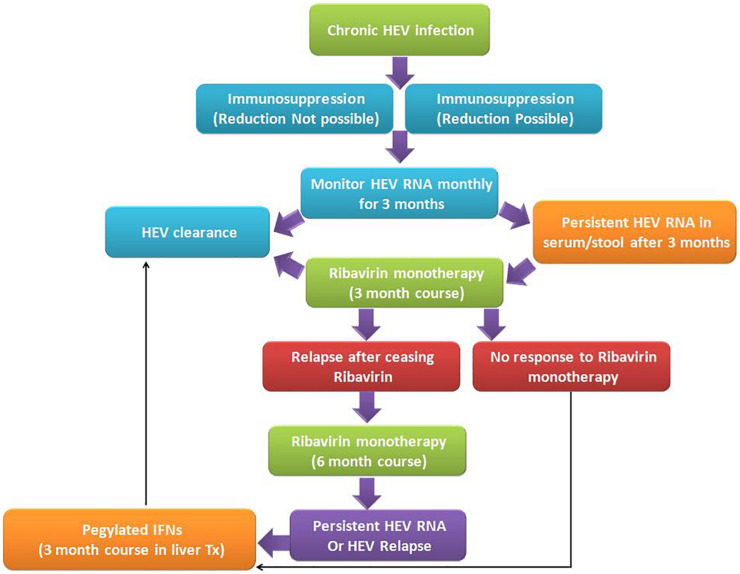
Algorithm for management of chronic hepatitis E viral infection: Chronic HEV infection in immunosuppressed patients is very common due to immunosuppressed state, wherever possible immunosuppressive doses need to be modulated as per the chronicity of the disease. However, in both the states, if HEV RNA persists for more than 3 months in serum and/or stool, ribavirin monotherapy for 3 months duration is the recommended treatment regimen. Such patients on treatment may be either non-responders to ribavirin therapy or clear the virus post-therapy. In a few cases, after ceasing the ribavirin therapy, relapse does occur, which is further treated with extended 6 months ribavirin monotherapy. Individuals who are non-responders or cases with HEV relapse, administration of pegylated interferon helps in the clearance of HEV viral infection and recovery of patients (Dalton et al., 2018).

So, based on the available data, the following algorithm should practice in the management and treatment of chronic hepatitis E (Figure 5), considering other clinical complications in the record too. First, screening for HEV RNA should be considered in immunocompromised patients, organ transplant recipients, and patients with unexplained hepatitis and cryptogenic cirrhosis to ascertain HEV infection. Second, a decrease in immunosuppression (if feasible) in transplant patients, to a level sufficient to allow for humoral and cellular immunity to curb HEV infection, should be the prime intervention in the management of chronic hepatitis E infection. Third, in the absence of adequate response, ribavirin therapy (600–800 mg/day for 3 months) should be the medication of choice. Fourth, in extreme cases, the HEV vaccine should be considered in patients undergoing immunosuppression as an effective treatment therapy.

## Conclusion

Since the discovery of the HEV in 1978, this has been neglected for a few decades and is considered as infection of developing countries only. However, in the last decade or so, an upsurge of HEV infections from around the world including developed nations too, classify HEV infection as the emerging which is now the prime health concern. Identification of new HEV genotypes, i.e., genotype-4, 5, 6, and, 7 in different animal reservoirs and hosts like boars, pigs, and camels from different geographical locations increases the mode and probability of cross-species infection. In addition to this, blood transfusion, organ transplants, and vertical mode of transmission also increased the infection risk. However, normally the course of infection is self-limiting, but recently, more aggressive chronic mode of infections have been reported in immunocompromised patients especially SOT recipients on immunosuppressive therapy. Initially, such chronic cases are predominant with HEV genotype-3, but now, genotype- 1, 2, and 4 also contributing, there worsening the situation. Patients with pregnancy or with dual viral infections like HIV-HEV or with hematological malignancies are also more prone to contact HEV infection.

Identification and isolation of HEV from the different organs like kidney, heart, spleen, thyroid, and pancreas, etc., suggested the extrahepatic manifestations which further complicated the understanding of the pathogenetic mechanism of hepatotropic HEV. With lots of progress in the basic and clinical virology which allows the development of efficient and feasible cell culture and animal models, and with the declaration of EASL guidelines for the HEV management, we now have a good insight of HEV pathophysiology and well-recommended diagnostic and treatment algorithm for HEV infected chronic patients.

## Author Contributions

RKR: conceptualization and supervision. VT: methodology and writing an original draft. VT, RKR, SK, SKS, and IB: validation. RKR, VT, IB, and PT: formal analysis. RKR, VT, SK, SKS, IB, and PT: writing – review and editing. All the authors have read and agreed to publish this version of the manuscript.

## Conflict of Interest

The authors declare that the research was conducted in the absence of any commercial or financial relationships that could be construed as a potential conflict of interest.
